# 4-Chlorophenol Oxidation Depends on the Activation of an AraC-Type Transcriptional Regulator, CphR, in *Rhodococcus* sp. Strain YH-5B

**DOI:** 10.3389/fmicb.2018.02481

**Published:** 2018-10-23

**Authors:** Hui Zhang, Ting Yu, Yiran Wang, Jie Li, Guangli Wang, Yingqun Ma, Yu Liu

**Affiliations:** ^1^College of Life Sciences, Huaibei Normal University, Huaibei, China; ^2^Advanced Environmental Biotechnology Centre, Nanyang Environment & Water Research Institute, Nanyang Technological University, Singapore, Singapore; ^3^School of Civil and Environmental Engineering, Nanyang Technological University, Singapore, Singapore

**Keywords:** 4-chlorophenol degradation, gene cluster, *Rhodococcus* sp. strain YH-5B, 4-chlorophenol monooxygenase, AraC-type transcriptional regulator

## Abstract

4-Chlorophenol (4-CP) oxidation plays an essential role in the detoxification of 4-CP. However, oxidative regulation of 4-CP at the genetic and biochemical levels has not yet been studied. To explore the regulation mechanism of 4-CP oxidation, a novel gene cluster, *cphRA2A1*, involved in biodegradation of 4-CP was identified and cloned from *Rhodococcus* sp. strain YH-5B by genome walking. The sequence analysis showed that the *cphRA2A1* gene cluster encoded an AraC-type transcriptional regulator and a two-component monooxygenase enzyme, while quantitative real-time PCR analysis further revealed that *cphR* was constitutively expressed and positively regulated the transcription of *cphA2A1* genes in response to 4-CP or phenol, as evidenced by gene knockout and complementation experiments. Through the transcriptional fusion of the mutated *cphA2A1* promoter with the *lacZ* gene, it was found that the CphR regulator binding sites had two 15-bp imperfect direct repeats (TGCA-N_6_-GGNTA) at −35 to −69 upstream of the *cphA2A1* transcriptional start site. Notably, the sub-motifs at the −46 to −49 positions played a critical role in the appropriate interaction with the CphR dimer. In addition, it was confirmed that the monooxygenase subunits CphA1 and CphA2, which were purified by His-tag affinity chromatography, were able to catalyze the conversion of 4-CP to 4-chlorocatechol, suggesting that strain YH-5B could degrade 4-CP via the 4-chlorocatechol pathway. This study enhances our understanding of the genetic and biochemical diversity in the transcriptional regulation of 4-CP oxidation in Gram-positive bacteria.

## Introduction

Chlorophenols are important building blocks for the manufacturing of lumber preservatives, antioxidants, pesticides, herbicides, and other industrial chemicals (Tobajas et al., 2012). These compounds are introduced into the environment as a result of anthropogenic activity such as industrial release, agricultural use, and waste incineration (Monsalvo et al., 2009). Due to their acute toxicity and carcinogenicity, the U.S. Environmental Protection Agency has listed chlorophenols as priority pollutants (Crosby, 1981). 4-Chlorophenol (4-CP) is the most common monochlorophenol isomer, and has been shown to be more toxic than either 2-chlorophenol or 3-chlorophenol (Caldeira et al., 1999). To date, several microorganisms have been isolated due to their ability to utilize 4-CP as a sole carbon and energy source (Finkelǐshtein et al., 2000). Two major pathways, namely the hydroquinone pathway (Bae et al., 1996) and the 4-chlorocatechol (4-CC) pathway (Radianingtyas et al., 2003), have been proposed for the bacterial degradation of 4-CP based on the different catabolic intermediates. In the hydroquinone pathway, 4-CP is first converted to hydroquinone following the release of a chloride ion, and is then transformed to 1,2,4-benzentriol, which is susceptible for *ortho* cleavage of its aromatic ring (Bae et al., 1996; Cho et al., 1998). In the 4-CP pathway, 4-CP is first catalyzed to 4-CC, which subsequently undergoes *ortho*- (Solyanikova and Golovleva, 2004) or *meta*-ring cleavage (Hollender et al., 1997). Of note, *Arthrobacter chlorophenolicus* A6 has also been reported to degrade 4-CP either via the 4-CC pathway with 1,2,4-benzentriol as the ring cleavage substrate or via the hydroquinone pathway (Nordin et al., 2005). In both of these pathways, initial degradation apparently occurred with the hydroxylation of the 4-CP aromatic ring by a monooxygenase.

It has been reported that chlorophenol monooxygenase, which belongs to the family of Group D flavoprotein monooxygenases, can hydroxylate 2,4,5-trichlorophenol to 2,5-dichloro-p-hydroquinone and 5-chlorohydroxyquinol (Xun, 1996; Gisi and Xun, 2003; van Berkel et al., 2006). Using NADH, the small subunit reduces FAD to form FADH_2_, and the large subunit subsequently utilizes FADH_2_ to catalyze the oxidation of the substrate (Nordin et al., 2005). *Arthrobacter chlorophenolicus* A6 has been shown to degrade high concentrations (up to 350 mg L^−1^) of 4-CP (Backman and Jansson, 2004), likely mediated by the products of the *cph* gene cluster (Nordin et al., 2005). Sequence analysis revealed that CphC-I and CphB from *A. chlorophenolicus* A6 also belonged to the Group D flavoprotein monooxygenases, with 72.0 and 37.1% similarity to the two-component monooxygenase NpcA and NpcB of *Rhodococcus opacus* SAO101, respectively, suggesting that they are likely associated with the initiation of 4-CP degradation. In addition, the putative regulatory gene *cphR* in the MalT family of transcriptional regulators was found between c*phC-I* and c*phB* (Nordin et al., 2005). A similar gene organization has been reported in the *npd* gene cluster, which was involved in the 4-nitrophenol (4-NP) catabolism of *Arthrobacter* sp. strain JS443 (Perry and Zylstra, 2007). Recently, *cphC-I* and *cphB* genes from *A. chlorophenolicus* A6 were expressed in *E. coli* and the corresponding enzymes were characterized for the degradation of 4-CP (Kang et al., 2017).

Nevertheless, the oxidative regulation of 4-CP at the genetic and biochemical levels has not yet been studied. Therefore, this study aims to explore the regulation mechanism of 4-CP oxidation by cloning a novel gene cluster, *cphRA2A1*, known to be involved in 4-CP oxidation by *Rhodococcus* sp. strain YH-5B. Based on the deduced amino acid sequence, the *cphA2A1* and *cphR* genes were proposed to encode a two-component monooxygenase and an AraC-type family regulatory protein, respectively. Transcription activity of *cphA2A1* genes was analyzed through construction of mutants, in which the *cphR* gene was disrupted by inserting a tetracycline resistance gene. Promoter activity assays were performed to determine the sites in the *cphA2A1* promoter for interaction with the transcriptional regulator CphR.

## Materials and Methods

### Strains, Plasmids, Media, and Chemicals

Using 4-CP as the sole carbon source, the 4-CP-degrading bacterium *Rhodococcus* sp. YH-5B was isolated after five rounds of enrichment from 4-CP-contaminated soil from Hangzhou Qingfeng Chemical Co., Ltd. (Hangzhou, China). The bacterial strains and plasmids used in this study are listed in Table 1. *Rhodococcus* strains were grown in Luria-Bertani (LB) or mineral salts (MM) media (Zhang et al., 2016) with 0.3 mM 4-CP as the sole carbon source at 30°C and *E. coli* strains were grown in LB medium at 37°C. Where appropriate, antibiotics and other supplements were used at the following concentrations: ampicillin (100 mg L^−1^), kanamycin (50 mg L^−1^), tetracycline (10 mg L^−1^), and isopropyl β-D-1-thiogalactopyranoside (IPTG) (0.1 mM). All chemicals used were of analytical grade or higher purity and purchased from Aladdin Industrial Inc., China, unless otherwise stated. The oligonucleotides used in this study are summarized in Table 2.

**Table 1:** Bacterial strains and plasmids used in this study.

Strain or plasmid	Description	Reference or source
***Rhodococcus* sp. strains**		
YH-5B	4-CP-degrading bacterium, wild type	This study
YH-5B*ΔcphR*	YH-5B mutant with *cphR* gene disrupted	This study
YH-5B*ΔcphRC*	YH-5B*ΔcphR* strain with *cphR* gene complemented by plasmid pK*cphRC*	This study
JT-3	4-CP^−^ *lacZ*^−^ Streptomycin^r^	Zhang et al., 2015
***E. coli strains***		
BL21 (DE3)	F^−^ *ompT hsdS*(r_B_^−^m_B_^−^) *gal dcm lacY1* (DE3)	Novagen
S17-1	*thi pro hsdR hsdM^+^ recA* R^−^ M*^+^* RP4-2-Tc::Mu-Km::Tn*7*	Simon et al., 1983
**Plasmids**		
pK18*mobsacB*	Gene replacement vector derived from plasmid pK18; Mob^+^ *sacB*^+^ Km^r^	Schafer et al., 1994
pK4	*E. coli–Rhodococcus* shuttle vector, Km^r^	Hashimoto et al., 1992
pER-*lacZ*	*E. coli*–*Rhodococcus* promoter-probe vector, promoter-less *lacZ* as reporter, Tc^r^	provided by Dr. Mengya Li of Jiangnan University
pET-29a (+)	Expression vector, Km^r^	Novagen
pBR322	Source of tetracycline resistance gene	Bolivar et al., 1977
pET-*cphA1*	pET-29a (+) expressing *cphA1* gene	This study
pET-*cphA2*	pET-29a (+) expressing *cphA2* gene	This study
pET-*cphR*	pET-29a (+) expressing *cphR* gene	This study
pK*cphRud*	pK18*mobsacB* carrying the flanking regions of *cphR* gene	This study
pK*cphR-tet*	pK18*mobsacB* carrying the flanking regions of *cphR* gene disrupted by *tet* gene	This study
pK4*cphRC*	pK4 carrying the entire *cphR* gene and its putative promoter region	This study
pER-P_A2A1_*lacZ*	pER-*lacZ* carrying the *cphA2A1* promoter	This study
pER-Pm78l*acZ*	pER-P_A2A1_*lacZ* with GCGA to TATC mutation from −75 to −78 upstream of the TSS	This study
pER-Pm69*lacZ*	pER-P_A2A1_*lacZ* with TGCG to GTAT mutation from −75 to −78 upstream of the TSS	This study
pER-Pm64*lacZ*	pER-P_A2A1_*lacZ* with AATC to CCGA mutation from −75 to −78 upstream of the TSS	This study
pER-Pm59*lacZ*	pER-P_A2A1_*lacZ* with GGAT to TTCG mutation from −75 to −78 upstream of the TSS	This study
pER-Pm53*lacZ*	pER-P_A2A1_*lacZ* with CCA to AAC mutation from −75 to −78 upstream of the TSS	This study
pER-Pm49*lacZ*	pER-P_A2A1_*lacZ* with CGCG to ATAT mutation from −75 to −78 upstream of the TSS	This study
pER-Pm44*lacZ*	pER-P_A2A1_*lacZ* with TGTA to GTGC mutation from −75 to −78 upstream of the TSS	This study
pER-Pm39*lacZ*	pER-P_A2A1_*lacZ* with GGAT to TTCG mutation from −75 to −78 upstream of the TSS	This study

**Table 2:** Primers used in this study.

Primer purpose and name	Sequence (5′ to 3′)	Restriction site
**Cloning fragment of *cphA1* gene**		
cphfA1-F	GAYGAYGTCACCACTCAYCC	—
cphfA1-R	ATGAGTCCGGCRTCCGTYTC	—
**Expression plasmid construction**		
cphR-F	TTGGAATTCATGATCGGTACA GCTCCCGG	*Eco*RI
cphR-R	TTTAAGCTTGCGCCTCAACA GCTCGGAA	*Hin*dIII
cphA1-F	TTTGGATCCATGACCACCTAC GAAATCC	*Bam*HI
cphA1-R	TTTGCGGCCGCCGTCTTCG CGAAGGAGCGC	*Not*I
cphA2-F	GGGGAATTCATGGATCCCAAT CAGTTCCGA	*Eco*RI
cphA2-R	TTTCTCGAGGATGGTCTGCG GTCCTGG	*Xho*I
**Disruption**		
cphRd-F	TTGAGGCCTAGCGGAGATGG TGGAGGTGGCT	*Stu*I
cphRd-R	TTTGAATTCCGACGATGGCG CATTCCGTAC	*Eco*RI
cphRu-F	TTGAAGCTTAATGTGGCTGG TCACGGGTTTG	*Hin*dIII
cphRu-R	TTTAGGCCTCGAAGATCGAC CTCATCAGGGTA	*Stu*I
Tet-F	TAACGCAGTCAGGCACCGTGT	—
Tet-R	GTTAGCGAGGTGCCGCCGGCT	—
cphRC-F	TTTGAATTCGCCAGGATGTTC ACGGCAAAGG	*Eco*RI
cphRC-R	TTTGAATTCGAGGATCTGGAA GAGGGCCTGATTT	*Eco*RI
**qRT-PCR**		
QcphR-F	ACCGAGTTGGGACCGCTGAGGA	—
QcphR-R	TGGACATACCGTCGCAACACCCT	—
QcphA2-F	GCCAAGGCGCAGACGACCAA	—
QcphA2-R	AAGGGACCGTTCGAGCCAAGC	—
Qtet-F	CCGGGCCTCTTGCGGGATAT	—
Qtet-R	GCTCCAAGTAGCGAAGCGAGCAG	—
Q16S-F	CGGTTTGTCGCGTCGTTTG	—
Q16S-R	GCTTTCGTTCCTCAGCGTCAGT	—
**5′ RACE**		
cphR-GSP1	GTCGGGTGGACATACCGTCGCAAC	—
cphR-GSP2	CACCGACAAGAGGTCACCGGAGAG	—
cphA2-GSP1	CTCCAGTGCCGTCGATCCACCGTG	—
cphA2-GSP2	AAGCTCATCGCGGTGTCAGCCTGG	—

### Cloning of *cphRA2A1* Gene Cluster and Sequence Analysis

It has been reported that the 4-NP monooxygenase from *Rhodococcus* sp. PN1 is able to catalyze the hydroxylation of both 4-CP and 4-NP (Takeo et al., 2008). A cphfA1-F/R primer pair (Table 2) was designed based on the conserved region of the oxygenase component of 4-NP monooxygenase from *Rhodococcus* sp. strain PN1 (accession no. AB081773) and used to amplify a putative 4-CP monooxygenase encoding gene from strain YH-5B. The resulting PCR product was sequenced and the *cphRA2A1* gene cluster was obtained by the genome walking method (Siebert et al., 1995). Open reading frames (ORFs) were identified using the ORF Finder online program at the NCBI website. Multiple sequence alignments were performed using CLUSTAL W and exported using the MEGA 6.0 software with the neighbor-joining method (Zhang et al., 2017).

### Plasmid Construction

The genomic DNA of strain YH-5B was extracted by high-salt precipitation (Zhang et al., 2016). To construct expression plasmids in *E. coli*, the *cphA1*, *cphA2*, and *cphR* genes were amplified with the primer pairs cphA1-F/A1-R, cphA2-F/A2-R, and cphR-F/R-R, respectively, using genomic DNA from strain YH-5B as a template. The PCR products were cloned into the plasmid pET-29a (+) with a C-terminal 6 × His tag, yielding plasmids pET-*cphA1*, pET-*cphA2*, and pET-*cphR*, respectively. All procedures resulted in a C-terminal His-tagged fusion protein.

For construction of the *cphR* knockout strain, plasmids with sequences up- and downstream of *cphR* were constructed using the primer pairs cphRd-F/Rd-R and cphRu-F/cphRu-R, respectively. The PCR products were successively cloned into the vector pK18*mobsacB* (Schafer et al., 1994), yielding plasmid pK*cphRud*. The tetracycline resistance gene (*tet*) (with its native ribosome-binding sequence) was amplified using the primer pair Tet-F/Tet-R and plasmid pBR322 (J01749) (Bolivar et al., 1977) as the template and then inserted into pK*cphRud* in the same transcriptional direction as *cphR* to yield plasmid pK*cphR-tet* for disruption of *cphR*. This plasmid was then introduced into the *E. coli* strain S17-1 (Simon et al., 1983), followed by conjugation with strain YH-5B (Saltikov and Newman, 2003). The mutant strain YH-5BΔ*cphR*, in which the double crossover event has occurred, was screened on LB plates supplemented with 10% (w/v) sucrose and tetracycline and the knockout of *cphR* was confirmed by PCR and sequencing (Min et al., 2016). The plasmid pK4*cphRC* for *cphR* complementation was constructed by cloning the entire *cphR* gene together with the putative promoter region into the *E. coli–Rhodococcus* shuttle vector pK4 using the primer pair cphRC-F/R (Hashimoto et al., 1992). The resulting construct was then transformed by electroporation into the competent cells of the mutant strain YH-5BΔ*cphR* (Takeo et al., 2003), screened based on its resistance to kanamycin, and verified by PCR.

To construct plasmids for promoter activity assays, the wild-type and mutated *cphA2A1* promoters bearing a variety of random substitutions from −35 to −78 bp upstream of the transcriptional start site (TSS) were synthesized and cloned into pER-*lacZ* (provided by Dr. Mengya Li of Jiangnan University, China). Each of the yielding relevant plasmids, in which all the promoters and *lacZ* were transcribed in the same direction, was introduced into the surrogate host *Rhodococcus* sp. strain JT-3 (Zhang et al., 2017) (Table 1) together with the plasmid pK*cphRC*. The strain JT-3, harboring pER-*lacZ*-derived plasmids and pK*cphRC*, was grown on LB plates containing kanamycin and tetracycline and confirmed as above.

### Quantitative Real-Time RT-PCR (qRT-PCR)

*Rhodococcus* sp. strain YH-5B and its mutants were grown in MM containing 0.2% yeast extract to an optical density at 600 nm (OD_600_) of 0.3. After inducing with 0.1 mM 4-CP for 3 h, cells were harvested and washed twice with PBS buffer (50 mM, pH7.5) before freezing at −70°C. Cells were then subjected to vigorous shaking using glass beads (size 10). Total RNA from strain YH-5B and its mutants was isolated using the RNAiso Plus according to manufacturer guidelines (TaKaRa, Dalian, China) and treated with gDNA Eraser (TaKaRa) to remove DNA contamination. First strand cDNA was synthesized from 1 μg of total RNA in a 20 μL reaction mixture using the PrimeScript RT reagent kit according to the manufacturer’s instructions (TaKaRa). Prior to RT-PCR assays, the cDNA templates were diluted to a final concentration of 0.8 ng μL^−1^. RT-PCR was performed on the ABI 7500 Fast Real Timer PCR system using SYBR Premix Ex Taq II (TaKaRa). The specific primer pair concentration (Table 2) was 300 nM and 1 μL of cDNA was added to 25 μL of the reaction mixture. The 16S rRNA gene from strain YH-5B was used as a reference to normalize the relative abundance of transcripts. The amplification efficiencies of each primer pair were between 92.4 and 98.2%, which is acceptable for a reliable real-time PCR quantification. All qRT-PCR assays were performed using three technical replications. The average C_T_ number of each triplicate RT-PCR reaction was used in statistical analyses. The 2^−ΔΔC^_T_ method was used to calculate the relative transcriptional levels of all the genes detected (Livak and Schmittgen, 2001).

### β-galactosidase Assays

Steady-state β-galactosidase assays were performed to detect the expression of promoter-*lacZ* fusion in strain JT-3, which is incapable of degrading 4-CP. Strain JT-3, bearing a pER-*lacZ* derived promoter test plasmid and pK*cphRC*, was grown overnight in LB medium containing the appropriate antibiotics. Cells were then diluted 100-fold in fresh LB with or without 4-CP and cultured for a further 4 h prior to harvesting. β-Galactosidase activity was measured in permeabilized cells in a 96-well plate as previously described (Griffith and Wolf, 2002).

### Protein Expression and Purification

The expression plasmids pET-*cphA1*, pET-*cphA2*, and pET-*cphR* were respectively transformed into *E. coli* BL21 (DE3) cells, grown in LB with kanamycin at 37°C to a turbidity of 0.5 at OD_600_, and then induced with IPTG for 16 h at 20°C. The cells were harvested by centrifugation, washed, and then suspended in PBS buffer (50 mM, pH7.5), followed by sonication. The clear supernatant was loaded onto a H60 Ni^2+^ Affinity Gravity Column according to the manufacturer’s instructions (TaKaRa). The target proteins were eluted with Elution Buffer having an increasing imidazole gradient of 20 to 250 mM. Finally, 1 mL fractions were collected and analyzed by SDS-PAGE, and those that contained the target protein were pooled and desalted by gel filtration on a PD-10 column (GE Healthcare, China) pre-equilibrated with phosphate buffer (50 mM, pH7.5) to remove excess imidazole. Protein concentrations were determined using the Bradford assay (Zhang et al., 2016).

### Enzyme Assays

The monooxygenase activity of H_6_-CphA1 and H_6_-CphA2 was determined by analyzing the decrease in substrate concentrations as determined by high-performance liquid chromatography (HPLC). The reaction mixture contained 200 μM NADH, 25 μM FAD, 1 μg H_6_-CphA2, and variable amounts of H_6_-CphA1 (10–100 μg) in 1 mL of phosphate buffer (50 mM, pH 7.5). The reaction was started by the addition of 30 μM of substrates. One unit (U) of activity was the amount of enzyme needed to catalyze the conversion of 1 μmol of substrates per min. Three independent sets of experiments were performed with at least six substrate concentrations ranging from 0.5 to 4 *K*_m_.

### Analytical Methods

The above reaction mixtures for enzyme assays were extracted twice with an equal volume of ethyl acetate. The organic layer was recovered, dried, and re-dissolved in 1 mL of methanol and subjected to HPLC analysis (Agilent 1200, Agilent technologies, Santa Clara, CA, United States) equipped with a Zorbax C-18 ODS Spherex column (250 mm × 4.6 mm). The mobile phase was methanol and water (60:40, v/v) at a flow rate of 1.0 mL min^−1^ at 30°C. The absorption spectra from 260 to 310 nm were detected with an Agilent G1314A UV detector. The substrate and putative metabolite concentrations were determined based on the peak area from the calibration curve. For unambiguous identification of 4-CP metabolites catalyzed by *cphA2A1* genes encoding monooxygenase, LC-MS was performed with an Agilent Technologies 6300 Series liquid chromatography ion-trap mass spectrometer (LC-ITMS) (Santa Clara, CA, United States). The column and elution process were as same as those mentioned above for HPLC. The samples were ionized by electrospray in a negative polarity mode.

### Gel Filtration Chromatography

The native molecular mass of the purified proteins was determined by gel filtration chromatography using a Superdex 200 10/300 GL column (GE Healthcare, China) according to the manufacturer’s instructions. The column was pre-equilibrated and eluted with Tris-HCl buffer (50 mM, pH 7.5) containing 0.15 M NaCl at a flow rate of 0.5 mL min^−1^, which was controlled by an ÄKTA Purifier 10 system (GE Healthcare, China). The standard proteins used for calculating the native molecular mass of enzymes were carbonic anhydrase (30 kDa), bovine serum albumin (67 kDa), alcohol dehydrogenase (150 kDa), catalase (200 kDa), ferritin (440 kDa), and thyroglobulin (669 kDa).

### 5′ Rapid Amplification of cDNA Ends (5′RACE)

The TSSs of the *cphA2A1* and *cphR* operons were determined by a RACE using the SMARTer RACE 5′/3′ Kit (TaKaRa). Total RNA was extracted using the RNAiso Plus (TaKaRa, Dalian, China) from strain YH-5B grown and induced with 4-CP in MM. The synthesis of first-strand cDNA was performed with random primers using the SMARTScribe Reverse Transcriptase, in which the latter added a few non-templated nucleotides to the 3′ end of the first-strand cDNA by its terminal transferase activity when reaching the 5′ end of the mRNA template. The SMARTer II A Oligonucleotide (TaKaRa) containing a terminal stretch of modified bases was then annealed to the tail of the cDNA and served as an extended template for SMARTScribe Reverse Transcriptase, thereby generating a tailed cDNA copy of the original RNA with the additional SMARTer sequence at the end. The primary RACE PCR products were amplified using the tailed cDNA as a template with the primer pairs Universal Primer A Mix (UPM) (TaKaRa)/cphA2-GSP1 and UPM/cphR-GSP1. Finally, nested PCR was performed with the primer pairs UPM/cphA2-GSP2 and UPM/cph-GSP2 using an aliquot of the primary RACE PCR products as a template. The final PCR products were cloned into the pMD18-T vector as per the manufacturer’s instructions (TaKaRa) and sequenced.

### Nucleotide Sequence Accession Number

The nucleotide sequences of an approximately 4.2 kb DNA fragment harboring the *cphRA2A1* gene cluster from *Rhodococcus* sp. strain YH-5B described in this study has been deposited in GenBank under accession no. MH129617.

## Results and Discussion

### Cloning and Sequence Analysis of the *cphRA2A1* Gene Cluster

**FIGURE 1:**
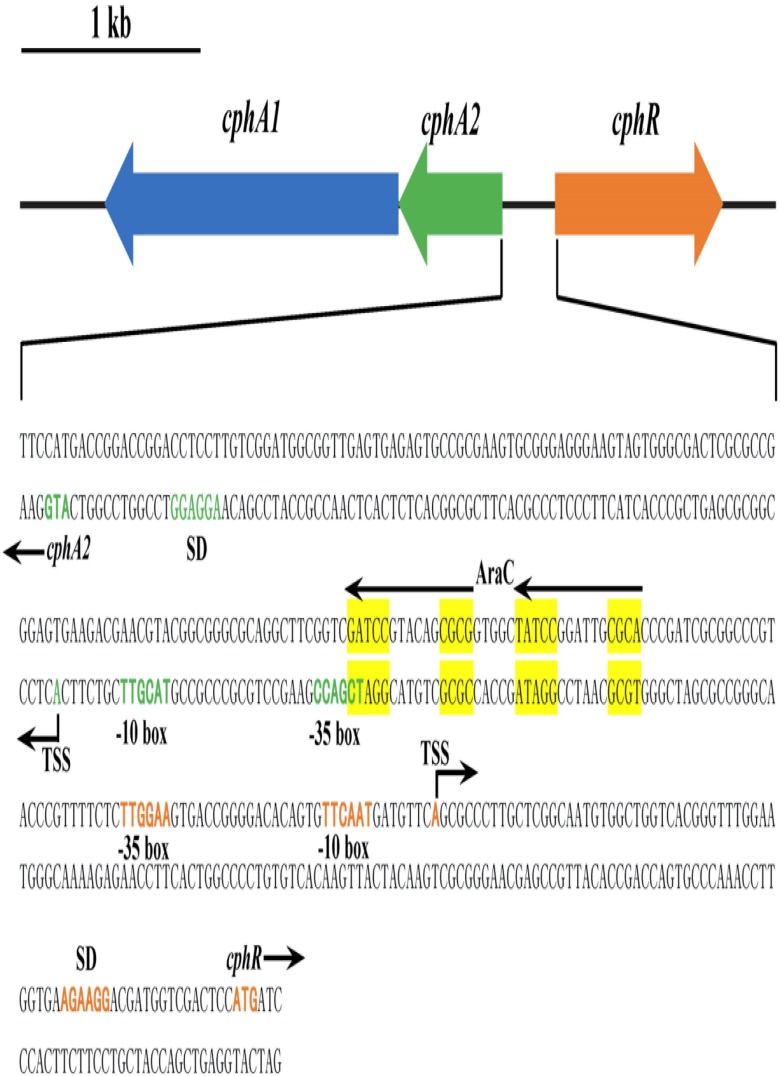
Scheme of the *cphRA2A1* gene cluster and the intergenic region between *cphR* and *cphA2* in *Rhodococcus* sp. strain YH-5B. The putative –10 box, –35 box, Shine-Dalgarno (SD) sequence, the proposed translational start codon ATG, and the determined transcriptional start sites (TSSs) of *cphR* (orange) and *cphA2* (green) are in bold. The possible AraC-type CphR regulator binding sites (RBSs) (TGCA-N6-GGNTA) are shown by the two arrows and tandemly imperfect direct repeats are shaded in yellow.

The putative 4-CP monooxygenase encoding gene was amplified from genomic DNA of strain YH-5B, yielding a 476 bp DNA fragment. The flanking region of this PCR product was obtained by genome walking. Sequence analysis revealed that the 4217 bp DNA fragment harbored three complete ORFs, designated as *cphR*, *cphA2*, and *cphA1* (Figure 1). As a typical representative of the Group D flavoprotein monooxygenases, the 4-hydroxyphenylacetate 3-monooxygenase system (*hpaBC*) of *E. coli* strain W ATCC 11105 exhibited similar genetic organization to *cphRA2A1* and also possessed the regulatory gene *hpaA* encoding an AraC-type regulatory protein (Prieto and Garciìa, 1997). Nevertheless, *hpaBC* and *hpaA* were transcribed in the same direction, *cphR* and *cphA2A1* transcription occurred diversely.

The deduced amino acid sequences of *cphA1* and *cphA2* showed 53 and 39% similarity with the corresponding component of 4-NP monooxygenase (BAB86378) from *Rhodococcus* sp. PN1, whereas they showed 38% and 39% similarity with those of putative chlorophenol monooxygenase (AAN08754) from *Arthrobacter chlorophenolicus* A6. In general, the reductase subunit has a flavin reductase domain and a C-terminal region with helix-turn-helix (HTH) motif, which has been described in the reductases of two-component monooxygenases involved in 4-CP (Nordin et al., 2005), 4-fluorophenol (Ferreira et al., 2009), and 4-NP (Takeo et al., 2003) catabolism. This HTH domain is expected to play an important role in substrate recognition and localization (Aravind et al., 2005). The deduced amino acid sequences of CphA1 and CphA2 revealed the presence of conserved N terminal 4-hydroxyphenylacetate 3-monooxygenase (HpaB, pfam11794) and flavin reductase-like regions (pfam01613), respectively. Based on the homology analysis as well as the analogy between the 4-CP and 4-NP degradation pathways, we deduced that *cphA1* and *cphA2* genes encode a two-component 4-CP monooxygenase belonging to the Group D flavoprotein monooxygenases (Heine et al., 2018).

The *cphR* gene product (CphR) exhibited a significant amino acid similarity to proteins annotated as AraC family transcriptional regulators from *Rhodococcus enclensis* 23b-28 (71%, PCK27969) and *Gordonia bronchialis* DSM 43247 (43%, ACY21077), respectively. The amino acid sequences BLAST results revealed that the conserved HTH motif for DNA binding (COG2207) is present at the C-terminal domain of the CphR protein (amino acid 190 to 308). Similar regulatory genes have been reported in 4-CP (Nordin et al., 2005), 4-fluorophenol (Ferreira et al., 2009), and 4-NP degradation gene clusters (Perry and Zylstra, 2007; Takeo et al., 2008). The above sequence analysis suggested that genes *cphA1*, *cphA2*, and *cphR*, as a gene cluster, are likely involved in 4-CP oxidation; *cphA1* and *cphA2* encoded an oxygenase and a flavin reductase component of 4-CP monooxygenase, respectively, and *cphR* produced an AraC-type regulatory protein, regulating the expression of *cphA2A1*.

### Positive Regulation of CphR for 4-CP Hydroxylation

**FIGURE 2:**
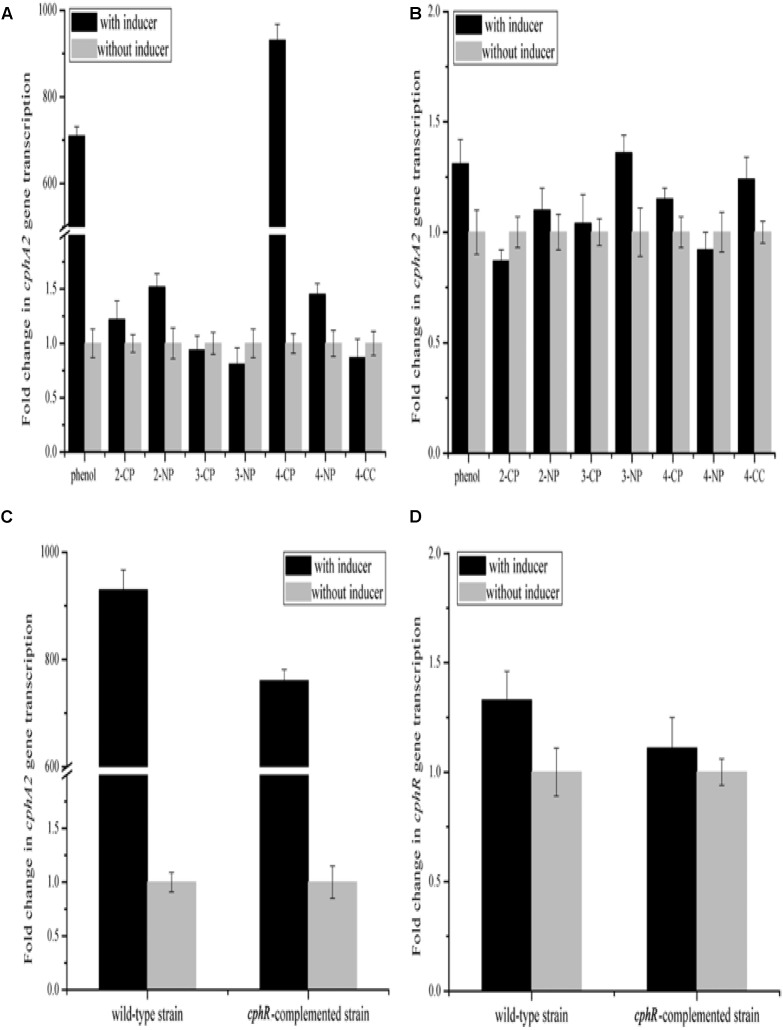
Transcriptional analyses of *cphA2A1* genes in the wild-type strain YH-5B **(A)** and the *cphR* knockout strain YH-5B*ΔcphR* **(B)** in the presence (black) or absence (LT gray) of various phenolic compounds. Transcriptional analyses of *cphA2A1* **(C)** and *cphR* **(D)** genes in the wild-type strain YH-5B and the *cphR*-complemented strain YH-5BΔ*cphRC* in the presence (black) or absence (LT gray) of 4-CP. The transcription activities of gene tested in each sample were calculated as the fold ratio following normalization to that of 16S rRNA gene.

YH-5B cells grown following induction by 4-CP for 5 h exhibited the ability to rapidly degrade 4-CP (0.3 mM) within 3 h; however, the cells grown with no pre-induction of 4-CP could not degrade 4-CP. This finding thus indicates that the expression of enzymes involved in 4-CP catabolism is most likely induced by 4-CP. We further measured the transcription activity of *cphA2A1* genes by qRT-PCR assays using various phenolic compounds as inducers. 4-CP or phenol increased the transcriptional levels of *cphA2A1* to 930- and 710-fold, respectively. In contrast, when 4-NP, 3-chlorophenol, 3-nitrophenol, 2-chlorophenol, 2-nitrophenol, or 4-CC were added as an inducer, the transcriptional levels of *cphA2A1* were similar to those without induction (Figure 2A). These findings evidently demonstrated that only two (4-CP and phenol) of the substrates tested were able to induce *cphA2A1* expression, though three substrates (4-NP, 3-chlorophenol, and 3-nitrophenol) could be hydroxylated by strain YH-5B following induction with 4-CP. Strain IF1 was able to utilize 4-fluorophenol or 4-NP, with both compounds functioning as inducers of expression of genes encoding monooxygenase (Ferreira et al., 2009). However, the expression of the *nphA1A2* gene of *Rhodococcus* sp. PN1 was found to be induced only by 4-NP among the several compounds tested in the presence of regulatory gene *nphR* (Takeo et al., 2003). A likely explanation for these variances is that there were significant differences in the specificity between enzymatic substrates and gene expression regulators. Thus, CphR in strain YH-5B is a specific transcriptional regulator rather than a global regulator and the 4-CP monooxygenase system is likely to differ from the previously reported 4-NP monooxygenase system.

To assess the effect of the putative transcriptional regulator CphR on 4-CP hydroxylation, we generated a mutant strain YH-5B*ΔcphR* in which *cphR* was replaced by the *tet* gene through double crossover recombination and the transcription of *tet* was controlled by the *cphR* promoter. Notably, the mutant strain YH-5B*ΔcphR* could not degrade 4-CP under any condition (data not shown) and the transcriptional levels of *cphA2A1* remained unaltered (Figure 2B). Since it was difficult for *cphR* to be recruited into the original locus of the mutant strain YH-5B*ΔcphR* genome through homologous recombination without introduction of a resistance gene, we employed the pK*cphRC* plasmid, which is capable of producing mature CphR protein, and transformed it into strain YH-5B*ΔcphR* to *trans*-complement *cphR* expression. The *cphR*-complemented strain YH-5B*ΔcphRC* regained the ability to degrade 4-CP in the presence of the inducer (data not shown). Moreover, the transcriptional levels of *cphA2A1* and *cphR* were found to be almost comparable in the complemented strain YH-5BΔ*cphRC* and the wild-type strain YH-5B under the same conditions (Figures 2C,D). Using this mutant strain, we then evaluated the effect of CphR on its own transcription. Importantly, the transcriptional level of *tet* in the *cphR*-complemented strain YH-5B*ΔcphRC* was found to be similar to that of *cphR* in wild-type strain YH-5B (Figure 2D). These results thus support the hypothesis that *cphR* encodes a positive regulatory protein for *cphA2A1* expression under 4-CP-induced conditions and that its transcription in strain YH-5B is constitutive. Degradation of 4-substituted phenols has been reported to be controlled by the LysR-type transcriptional regulators in most cases (Kitagawa et al., 2004; Yamamoto et al., 2011; Min et al., 2016), which positively regulate the expression of the target genes in the presence of the corresponding inducer. The AraC-type family regulators consist of more than 100 proteins controlling the transcription of genes involved in carbon metabolism, pathogenesis, and response to alkylating agents in bacteria (González-Pérez et al., 1999). In *Rhodococcus* sp. strain PN1, the AraC-type regulator was found to activate transcription of the *nphA1A2* gene encoding the two-component 4-NP monooxygenase and the *nphR* gene showed constitutive expression (Takeo et al., 2008). However, the LysR-type regulator PnpR, which is involved in the activation of 4-NP degradation in *Pseudomonas* sp. WBC-3, positively regulates its own synthesis (Zhang et al., 2015). Although the genetic make-up of the gene clusters under the control of AraC-type and LysR-type regulators are similar, their binding and recognition sequences differ (Zhang et al., 2015). Moreover, the gene clusters regulated by AraC-type proteins are generally induced by phenolic substrates, such as 4-NP (Takeo et al., 2008) and phenylacetate (Prieto and Garciìa, 1997), while for LysR-type regulators, the intermediates were found to function as inducers (Veselý et al., 2007).

To further understand the transcriptional regulation of the *cphRA2A1* gene cluster, the TSSs of *cphR* and *cphA2A1* genes were determined by 5′ RACE using total RNA isolated from strain YH-5B grown in the presence of 4-CP. The identified TSSs were located at 67 and 89 bp upstream of the putative translational start codon ATG of *cphR* and *cphA2A1* genes, respectively (Figure 1). The supposed −35 and −10 boxes of each promoter with the appropriate intervals (18 bp for *cphR* and 17 bp for *cphA2*) were detected in the upstream region of the TSSs. The deduced Shine–Dalgarno sequence is also shown in Figure 1.

### Expression and Purification of Proteins

**FIGURE 3:**
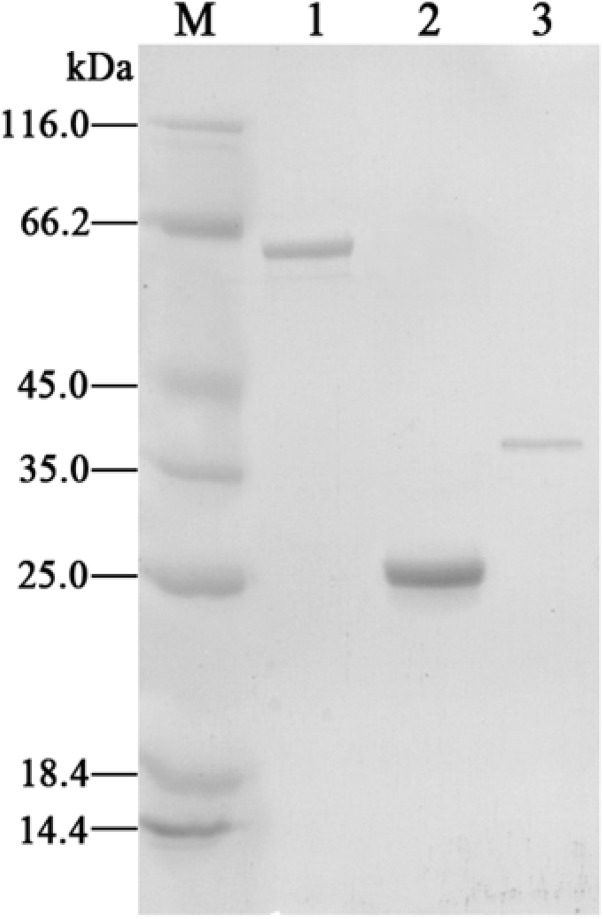
SDS-PAGE analyses of the purified H_6_-CphA1 (lane 1), H_6_-CphA2 (lane 2), and H_6_-CphR (lane3). Protein molecular mass standards are indicated in lane M.

To characterize the transcriptional regulator CphR and the putative 4-CP monooxygenase, we cloned the *cphR*, *cphA1*, and *cphA2* genes into the pET-29a (+) vector for expression as C-terminal His-tag fusion proteins. H_6_-CphA1 and H_6_-CphA2 proteins were soluble and readily purified, whereas H_6_-CphR was as well as largely produced as insoluble inclusion bodies, which is thought to be a characteristic property of the AraC-type family (Schleif, 2010). SDS-PAGE analyses of the purified H_6_-CphA1, H_6_-CphA2, and H_6_-CphR proteins showed single bands of approximately 65, 26, and 39 kDa, respectively, corresponding to the calculated molecular weight of the putative amino acid sequence (Figure 3). Gel filtration assay showed that the eluted H_6_-CphR in the presence or absence of 4-CP appeared as a single peak with a molecular mass of approximately 80 kDa (data not shown), thus indicating that CphR exists as a dimer. Notably, our finding is consistent with the literature describing the necessity of dimerization in AraC-type regulator proteins to occupy the binding sites and initiate transcription (Bustos and Schleif, 1993), whereas Lys-type regulators generally functions as tetramers (Zhang et al., 2015).

### Determination of CphR Binding Region for Activation of *cphA2A1* Promoter

**FIGURE 4:**
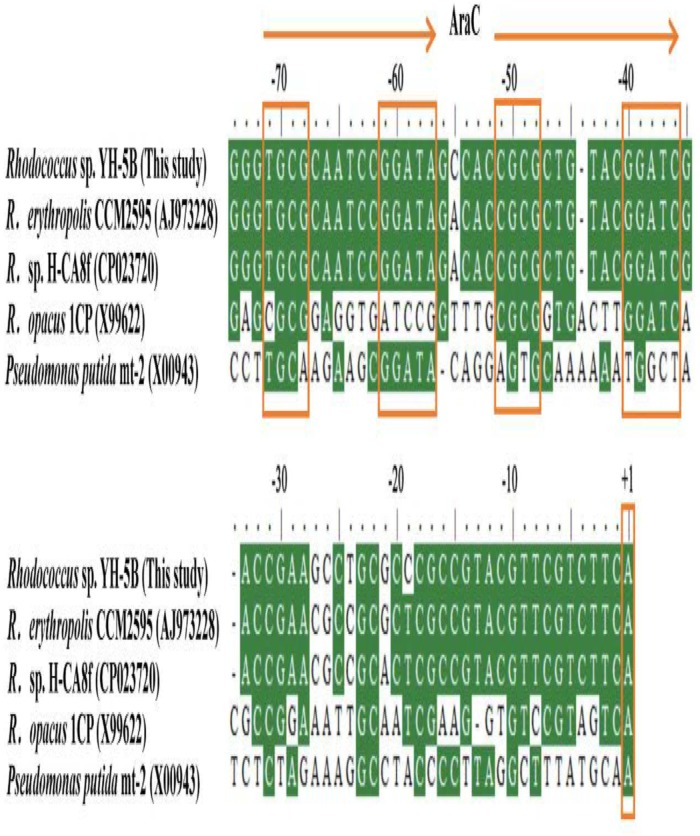
Multiple alignment of the putative regulation sequences in the intergenic region between *cphR* and *cphA2* of *Rhodococcus* sp. strain YH-5B and other related strains. The identified transcriptional start sites (TSSs) are indicated by +1 in box. The proposed two tandemly imperfect direct repeats for the AraC-type regulators binding are represented by the two arrows, in which each sub-motif is boxed.

**FIGURE 5:**
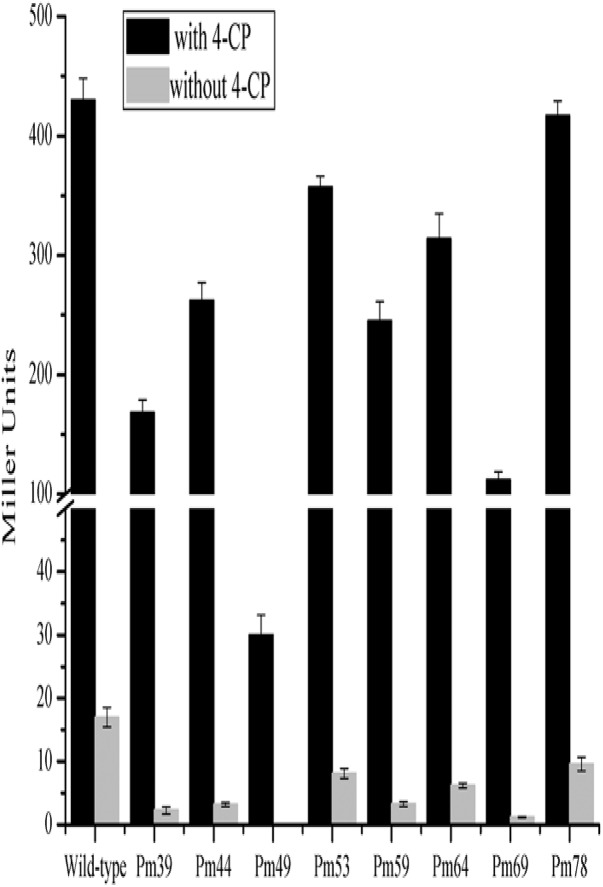
Determination of the promoter activities of the *cphA2A1* genes. The β-galactosidase activities were measured in the strain JT-3 carrying each pER-*lacZ* derived plasmid bearing mutated promoter and pK*cphRC* expressing CphR in the presence (black) or absence (LT gray) of 4-CP.

Multiple alignment analysis showed two homologous, 15-bp, tandemly imperfect direct repeats (TGCA-N_6_-GGNTA) in the promoter of *cphA2A1* (Figure 4), which is consistent with the consensus motifs of the AraC/XylS-type regulator binding sequences (RBSs) (González-Pérez et al., 1999). To determine the involvement of the proposed RBSs in *cphA2A1* promoter activation, we constructed eight plasmids with substitution mutations in the promoter region (Table 1). In the absence of 4-CP, the expression activity was very low or undetectable in strain JT-3 harboring each mutant promoter. Notably, in the presence of 4-CP, only the mutant promoter Pm49 exhibited < 10% of the wild-type activity, whereas five of the mutant promoters (Pm69, Pm64, Pm59, Pm44, and Pm39) showed reduced expression activity, varying between 74 and 27% (Figure 5). However, no or little significant effects were observed with the mutated promoter Pm53 and Pm78. These results suggest that the −46 to −49 region (CGCG) upstream of the *cphA2A1* TSS is critical for the CphR-dependent transcriptional activation of the *cphA2A1* promoter. The AraC/XylS family regulators have been reported to contain a highly conserved stretch (∼100 amino acids) with two possible HTH DNA binding motifs at the C-terminal region (Gallegos et al., 1997). The AraC-type protein consists of two monomers, each of which recognizes one of the tandem repeats (Reeder and Schleif, 1993). Each of the tandem repeats possesses two sub-motifs (5′ TGCA and 3′ GGNTA) separated by six bases and each sub-motif correspondingly interacts with one of the two HTH elements (Niland et al., 1996). The mutations of the −46 to −49 CGCG (Pm49) and the −66 to −69 TGCG (Pm69) sub-motifs abolish the transcription activity of the *cphA2A1* promoter, perhaps because CphR failed to appropriately interact with the sub-motif, thereby preventing the formation of the CphR dimer. The ability of the mutated promoter Pm39 to activate transcription was also affected, likely due to the incongruous interaction between the sub-motif and the HTH element leading to an unstable CphR dimer and thus affecting the interaction with RNA polymerase. Gallegos et al. speculated that the C-terminal domains of the AraC/XylS-type regulators carrying two possible HTH motifs are likely involved in the interaction with RNA polymerase (Gallegos et al., 1997; González-Pérez et al., 1999). Nevertheless, the tested N-terminal region of the RNA polymerase alpha subunit was proved to have no ability to interact with a variety of AraC/XylS family members for transcriptional activation (Egan et al., 2000). On the other hand, the −35 to −39 sub-motif overlaps by two bases with the −35 hexamer, which impairs the binding of RNA polymerase with the promoter. Additionally, the −75 to −78 region (GCGA) upstream of the TSS has been reported to be involved in the interaction with the HTH element of AraC/XylS family regulators as a sub-motif (Gallegos et al., 1997). However, this was not consistent with our results, suggesting that the region present in the *cphA2A1* promoter was likely not related to the activation of the CphR-dependent promoter. Taken together, the findings reveal that the CphR RBSs seems to be TGCA-N_6_-GGNTA, positioned at −35 to −49 and at −55 to −69 in the *cphA2A1* promoter.

### Characterization of the 4-CP Monooxygenase System

**FIGURE 6:**
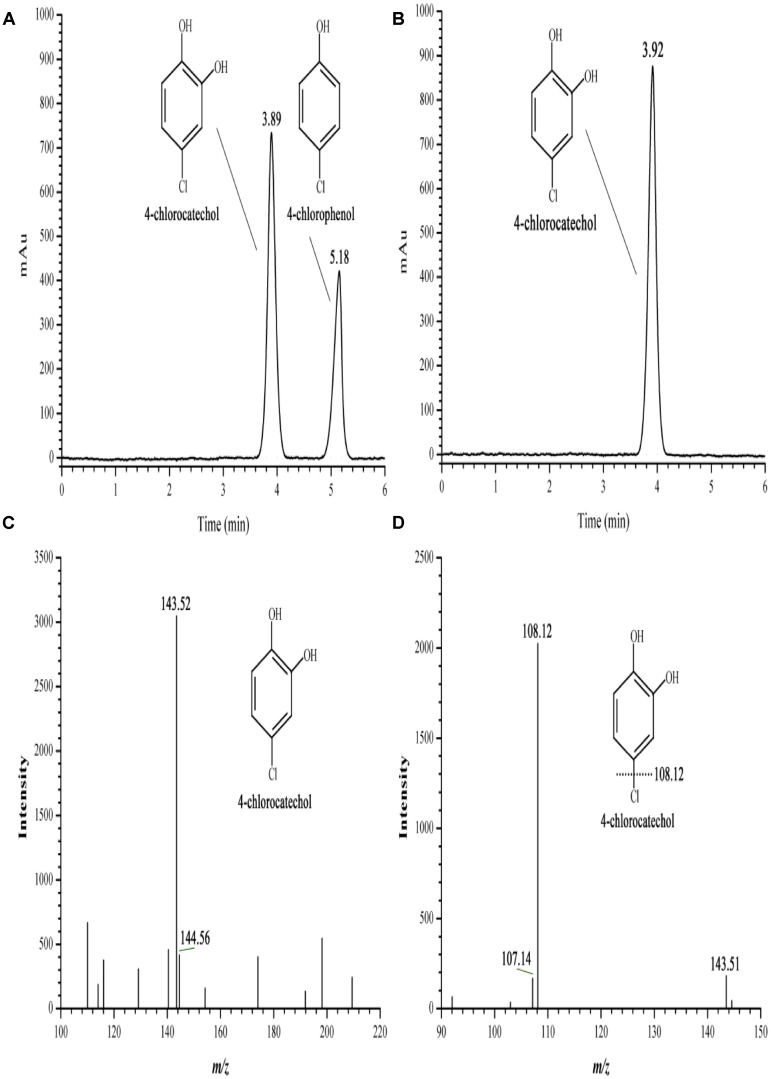
HPLC and LC-MS spectrum of the 4-CP conversion catalyzed by the two-component 4-CP monooxygenase consisting of H_6_-CphA1 and H_6_-CphA2. **(A,B)** HPLC of the 4-CP conversion and the standard 4-CC, respectively; **(C,D)** First and second-order mass spectrum of the 4-CP conversion, respectively.

Oxidation activity was measured in a reaction mixture composed of H_6_-CphA1, H_6_-CphA2, NADH, and FAD. In the HPLC chromatograms, the 4-CP conversion catalyzed by H_6_-CphA1 and H_6_-CphA2 had the same retention time as the standard 4-CC (Figures 6A,B). LC-MS analysis further confirmed that the two-component monooxygenase consisting of CphA1 and CphA2 in strain YH-5B was responsible for catalyzing the conversion of 4-CP to 4-CC (Figures 6C,D), thus demonstrating that strain YH-5B degrades 4-CP via the 4-CC pathway. The maximal degradation activity was achieved (0.0172 ± 0.0012 U mg^−1^) at a molar ratio of CphA2:CphA1 of approximately 1:86. The calculated *K*_m_ and *k*_cat_ values of the 4-CP monooxygenase were 8.7 ± 1.1 μM and 0.62 ± 0.04 min^−1^, respectively. The substrate specificity of the 4-CP monooxygenase was measured in the same reaction. This enzyme exhibited slightly lower activities toward 4-NP and phenol than toward 4-CP (Table 3). However, 4-hydroxyphenylacetate, 3-hydroxyphenylacetate, and 2-hydroxyphenylacetate were not oxidized by the two-component monooxygenase, nor were they able to induce the expression of *cphA2A1* (data not shown), suggesting that the 4-CP monooxygenase seemed to be completely different from 4-hydroxyphenylacetate 3-monooxygenase, although both monooxygenases have common conserved regions in their amino acid sequences.

**Table 3:** Kinetic parameters for the purified H_6_-CphA1 and H_6_-CphA2 toward phenolic compounds.

	*K*_m_(μM)	Enzyme activity (U mg^−1^)	Relative Enzyme activity (%)
phenol	9.6 ± 0.9	0.0167 ± 0.0015	97
4-chlorophenol	8.7 ± 1.2	0.0172 ± 0.0012	100
3-chlorophenol	47.7 ± 3.9	0.0105 ± 0.0009	61
2-chlorophenol	N.D.	N.D.	—
4-chlorocatechol	N.D.	N.D.	—
4-nitrophenol	12.1 ± 1.8	0.0161 ± 0.0017	94
3-nitrophenol	63.2 ± 7.7	0.0082 ± 0.0007	48
2-nitrophenol	N.D.	N.D.	—
4-hydroxyphenylacetate	N.D.	N.D.	—
3-hydroxyphenylacetate	N.D.	N.D.	—
2-hydroxyphenylacetate	N.D.	N.D.	—

*N.D. represents no or negligible activities (<0.001 U mg^−*1*^).*

## Conclusion

An approximately 4.2-kb 4-CP catabolic gene cluster was obtained from the newly isolated *Rhodococcus* sp. strain YH-5B. In this gene cluster, the *cphA2A1* and *cphR* genes encoded for a two-component monooxygenase, composed of CphA1 and CphA2, and an AraC-type transcriptional regulator CphR, respectively. CphR was expressed constitutively in strain YH-5B and able to activate the transcription of *cphA2A1* promoter in the presence of 4-CP or phenol. The −35 to −69 region upstream of *cphA2A1* TSS possesses a conserved AraC-type regulator, CphR RBSs and, importantly, a −46 to −49 sub-motif (CGCG) critical for the interaction with the dimer CphR. The purified H_6_-CphA1 and H_6_-CphA2 proteins exhibited broad substrate specificity and were responsible for the conversion of 4-CP to 4-CC. This study enhances our understanding of the genetic and biochemical diversity of the transcriptional regulation of 4-CP oxidation in Gram-positive bacteria.

## Author Contributions

HZ conceived and designed the experiments. HZ, TY, JL, and YW performed the experiments. HZ, GW, YM, and YL analyzed the data. HZ and GW wrote the manuscript. All authors reviewed the manuscript, and read and approved the final manuscript.

## Conflict of Interest Statement

The authors declare that the research was conducted in the absence of any commercial or financial relationships that could be construed as a potential conflict of interest.
